# Crystallization and Performance of Polyamide Blends Comprising Polyamide 4, Polyamide 6, and Their Copolymers

**DOI:** 10.3390/polym15163399

**Published:** 2023-08-14

**Authors:** Yajing Zhang, Mingda Wang, Di Zhang, Yibing Wang, Li Wang, Yongjun Qiu, Liquan Wang, Tao Chen, Liming Zhao

**Affiliations:** 1 State Key Laboratory of Bioreactor Engineering, East China University of Science and Technology, Shanghai 200237, China; zyjhy66@163.com (Y.Z.); mindawang@foxmail.com (M.W.); greatdedee@163.com (D.Z.); ybwang@ecust.edu.cn (Y.W.); li.wang@ecust.edu.cn (L.W.); qiuyongjun@ecust.edu.cn (Y.Q.); 2 Key Laboratory of Biobased Material Engineering, China National Light Industry, East China University of Science and Technology, Shanghai 200237, China; 3 Shanghai Collaborative Innovation Center for Biomanufacturing Technology (SCICBT), Shanghai 200237, China; 4 Shanghai Key Laboratory of Advanced Polymeric Materials, School of Materials Science and Engineering, East China University of Science and Technology, Shanghai 200237, China; lq_wang@ecust.edu.cn

**Keywords:** polyamide 4, PA4/PA6 blends, copolyamides 4/6, crystallization

## Abstract

Polyamide 4 (PA4) is a biobased and biodegradable polyamide. The high hydrogen bond density of PA4 bestows it with a high melting point that is close to its thermal decomposition temperature, thereby limiting the melt processing of PA4. In this study, PA4 was blended with polyamide 6 (PA6) and further modified with copolyamide 4/6 (R46). The effects of composition on the crystallization behavior of the blends were studied. The results demonstrated that the binary PA4/PA6 (B46) and ternary PA4/PA6/R46 (B46/R46) blends formed two crystalline phases (PA4- and PA6-rich phases) through crystallization-induced phase separation. With increasing PA6 content, the thermal stability and crystallinity of the B46 blend increased and decreased, respectively, and the contribution of PA6 toward the crystallization of the PA4-rich phase diminished. Molecular dynamics simulations showed the molecular chain orientation of the B46 blends well. The melting points, crystallinities, and grain sizes of the B46/R46 blends were lower than those of the B46 blends. The crystallization of the PA4-rich phase was restrained by the dilution effect of molten-state PA6, and the nucleation and crystallization of the PA6-rich phase were promoted by the presence of crystallized PA4. The B46 blends with 30–40 wt% PA6 had the best mechanical properties.

## 1. Introduction

Polyamide 4 (polybutyrolactam, PA4), which is synthesized using a biobased monomer, butyrolactam (2-pyrrolidone) [1], is the only environmentally friendly biodegradable polyamide (PA) [2,3,4] with broad application prospects. PA4 has a higher amide bond density and, consequently, a higher hydrogen bond density than other PAs. It therefore exhibits a high melting point and excellent mechanical properties [5]. The thermal degradation of PAs begins with the cleavage of the amide or methylene C–N bonds connected to amino groups [6]. Consequently, owing to its higher amide bond density, PA4 has a lower thermal decomposition temperature than other PAs. However, the processing temperatures of PAs with high melting points are high, and because the thermal decomposition temperature and melting point of PA4 are close to each other, the thermal processing of PA4 is challenging.

Blending is a convenient, effective, and low-cost method for improving the processability of polymeric materials and enhancing their properties [7]. Several PA blends have been reported, such as those with polypropylene [8], polyethylene [9], polyethylene terephthalate [10], polylactic acid [11], acrylonitrile butadiene styrene [12], and two types of PA [13,14,15]. Although most of these blends are immiscible, PA/PA blends are miscible. Certain PA/PA blends, such as PA410/PA6 [16], PA48/PA66 [17], PA6/PA66 [18], and PA56/PA66 [19], can even miscible in the crystalline phase. The PAs that form miscible blends have comparable average numbers of methylene units between their amide bonds.

The crystallization behavior has a critical influence on the properties of crystalline polymer blends [20]. PA11/PA610 blends are miscible only in the molten state [21] because crystallization-induced phase separation occurs upon cooling. Wide-angle X-ray diffraction (WAXD) experiments have demonstrated that pure PA11 and PA610 crystals form upon cooling PA11/PA610 blends, regardless of the blending ratio. The composition also affects the crystallization behavior of some crystalline polymer blends. Safari et al. [16] studied the composition-dependent miscibility of PA410/PA6 blends triggered by crystallization-driven phase separation. PA410/PA6 blends with 10 wt% or 20 wt% PA6 comprised a single PA410-rich crystalline phase; however, PA410/PA6 blends with >30 wt% PA6 underwent crystallization-induced phase separation, which gradually intensified with increasing PA6 content. This induced the formation of double-crystalline blends comprising PA6- and PA410-rich phases. Eventhough, research on PA/PA blends is quite scarce, especially for those involving PA4. Therefore, it is desirable to understand the crystallization behavior of PA/PA blends containing PA4.

On the other hand, PAs contain the same functional groups, which makes it difficult to distinguish the different molecular chains in PA/PA blends and limits microstructural observations. Molecular dynamics (MD) simulations therefore play a crucial role in microstructural analysis [22], understanding the crystallization mechanisms [23,24,25] and hydrogen bonding-induced phase changes in polymer blends [26].

In this study, to improve the processability of PA4, several PA4/PA6 binary blends (B46) were prepared using PA6 with a similar average number of methylene units between the amide bonds as that in PA4. In addition, because the addition of copolymers comprising blended components can improve the interfacial connectivity between homopolymeric components [27,28], PA4 and PA6 copolymers (random copolyamides 4/6, R46) were used to prepare several PA4/PA6/R46 ternary blends (B46/R46). The effects of the blending ratio and R46 composition on the crystallization behavior of the B46 and B46/R46 blends were investigated by WAXD, MD simulations, thermal analysis, and mechanical properties analysis. The results provide insight into how composition affects the crystallization behavior of PA4-based blends, which is expected to provide theoretical guidance for the melt processing of PA4.

## 2. Materials and Methods

### 2.1. Materials

PA4 (*M_n_* = 20,000) and R46 were prepared according to previously described methods [29]. PA6 (Novamid^®^ 1010C2; *M_n_* = 20,000) was supplied by DSM (Genk, Belgium). All PAs were dried using a vacuum drying oven (Yiheng Scientific Instrument Co., Ltd., Shanghai, China) at 80 °C for 48 h. The blends were prepared via melt mixing using a co-rotating twin-screw extruder (SJZS-10B, Ruiming Experimental Instrument Co., Ltd., Wuhan, China) at 260 °C. The compositions of the blends and R46 copolymers are summarized in Table 1 and our previous paper [29], respectively.

An SZS-20 reciprocating-screw injection molding machine (Ruiming Experimental Instrument Co., Ltd., Wuhan, China) was used to prepare injection-molded samples suitable for tensile testing (thickness = 2 mm, ISO 37:2005 [30], type 2). The melt and mold temperatures were set at 260 and 80 °C, respectively.

### 2.2. Differential Scanning Calorimetry (DSC)

Differential scanning calorimetry (DSC) was performed using a DSC-Q25 differential scanning calorimeter (TA Instruments, New Castle, DE, USA) to evaluate the thermal behavior of the blends. All experiments were performed under a nitrogen atmosphere at a flow rate of 40 mL min^−1^. The blends were vacuum-dried at 80 °C for 48 h prior to analysis. Each sample (5–8 mg) was first heated from 30 to 275 °C at a rate of 10 °C min^−1^ and maintained at 275 °C for 3 min to eliminate its thermal history, followed by cooling to 30 °C at a rate of 10 °C min^−1^.

### 2.3. Dynamic Mechanical Analysis (DMA)

Dynamic mechanical analysis (DMA; Q800, TA Instruments, New Castle, DE, USA) was used to measure the glass-transition temperature (*T*_g_) of each sample in single cantilever bending mode in the temperature range of −20 to 150 °C at a heating rate of 10 °C min^−1^. The dimensions of the test samples were 10 mm × 35 mm × 1.9 mm.

### 2.4. Thermogravimetric Analysis (TGA)

The thermal stability of the samples was analyzed via thermogravimetric analysis (TGA) using a TG209F1 instrument (NETZSCH-Gerätebau GmbH, Selb, Germany). Each sample was heated from 30 to 550 °C at a rate of 10 °C min^−1^ under a nitrogen atmosphere.

### 2.5. Wide-Angle X-ray Diffraction (WAXD)

The crystallization behavior of the blends was analyzed via WAXD using an 18 KW/D/max2550VB/PC instrument (Rigaku Industrial Corporation, Osaka, Japan). The methods and parameters are described in our previous paper [29].

### 2.6. Scanning Electron Microscopy (SEM)

The blend samples were frozen in liquid nitrogen. Subsequently, the samples were fractured, and the cryo-fractured surfaces were spray-coated with gold. Cross-sectional scanning electron microscopy (SEM) images were obtained at 25 °C using an S3400-N field emission scanning electron microscope (Hitachi Limited, Tokyo, Japan) at an accelerating voltage of 15 kV.

### 2.7. Mechanical Analysis

Tensile tests were performed using a 2T/CMT 4204 universal testing machine (Sans Testing Machine Co., Ltd., Shenzhen, China). The experiments and calculations of the tensile strength, fracture strain, and Young’s modulus were performed according to ISO 527-2:2012 [31].

### 2.8. Molecular Dynamics (MD) Simulations

The microstructures of the B46 blends were investigated through MD simulations; however, MD simulations were not performed for the B46/R46 blends owing to their complexity. Simulations were performed in a 50 × 50 × 50 *r*_c_^3^ periodic box using LAMMPS open-source software (stable_29Sep2021) [32]. Schematics of the PA4 and PA6 molecular chains and the molecular chain information for each blend are presented in Tables S1 and S2, respectively. A schematic of the hydrogen bonding interactions is shown in Figure S1. Details of the simulation methodology and coefficients are given in the Supplementary Materials [33,34,35,36,37,38].

## 3. Results and Discussion

### 3.1. Crystalline Properties

**Figure 1:**
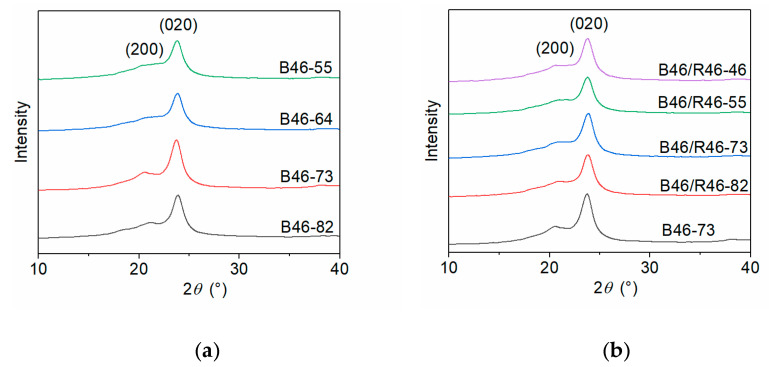
WAXD curves of the (**a**) B46 and (**b**) B46/R46 blends.

The crystallization parameters of the B46 and B46/R46 blends were determined using WAXD, and the WAXD curves are illustrated in Figure 1. The peak-differentiating and fitting results are shown in Figure S2 and summarized in Table 2. The strong diffraction peaks at the scattering angles 2*θ* ≈ 20.7° and 23.7° correspond to the (200) and (020) crystal planes of the blends. As the PA6 content increased, particularly between 40% and 50%, the (200) diffraction peak weakened, and the ratio of the areas of the diffraction peaks (*A*_1_/*A*_2_) decreased. In addition, the crystallinity and grain size of the blends decreased gradually. Increasing the PA6 content increases the number of long methylene chain segments in the B46 blends, which alters their conformation during crystallization and inhibits the growth of the (200) crystal plane. For the B46/R46 blends, the diffraction peaks of (200) and (020) crystal planes gradually weakened as the content of PA6 structural units (C6) increased, but the *A*_1_/*A*_2_ ratios did not differ significantly. All the B46/R46 blends had considerably smaller grains than B46-73. This indicates that the addition of R46 to B46 induced a more random distribution of amide bonds and hindered the crystallization of the molecular chains by breaking the regular arrangement of intermolecular hydrogen bonds. Consequently, the crystallinities of the B46/R46 blends were lower than that of B46-73.

**Figure 2:**
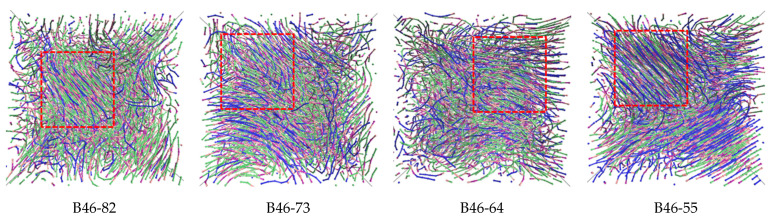
Molecular chain distribution after MD-simulated crystallization of the B46 blends with various compositions. Green: methylene group in PA4; blue: methylene group in PA6; pink: N atom in the amide group; purple: carbonyl group in the amide group. The red dotted boxes indicate regions of ordered alignment.

**Figure 3:**
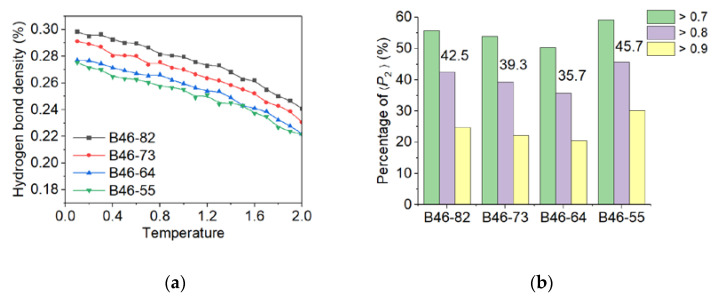
(**a**) Hydrogen bond density and (**b**) distribution of local orientation degree of the B46 blends. The numbers on the bar graph represent the crystallinity of each blend.

Polarizing microscope has been used to observe the crystallization process and structure. However, no meaningful result could be obtained due to thermal decomposition at high temperature. Alternatively, MD simulation was chosen to analyze the microstructure of the blends. The simulated distributions of molecules within the B46 blends are shown in Figure 2, and the calculated crystallinities (see Supplementary Materials) are shown in Figure 3. At 20–40% PA6, the ordered alignment of the molecular chains (regions within the red dotted boxes in Figure 2) gradually changed from a parallel tight alignment to a loose curved alignment; in addition, the crystallinity gradually decreased with increasing PA6 content, which agrees with the WAXD results. However, the MD simulations suggest that B46-55 (with 50% PA6) had the highest crystallinity, along with many parallel and closely aligned ordered regions. This may be because the MD simulations were performed under relatively ideal conditions, in which the hydrogen bond density played a key role in the crystallization process. Of the B46 blends, B46-55 had the lowest hydrogen bond density and a smaller free-motion confinement effect on the molecular chains, which was more favorable for the ordered arrangement of the molecular chains.

### 3.2. Thermal Properties

#### 3.2.1. Thermomechanical Properties

**Figure 4:**
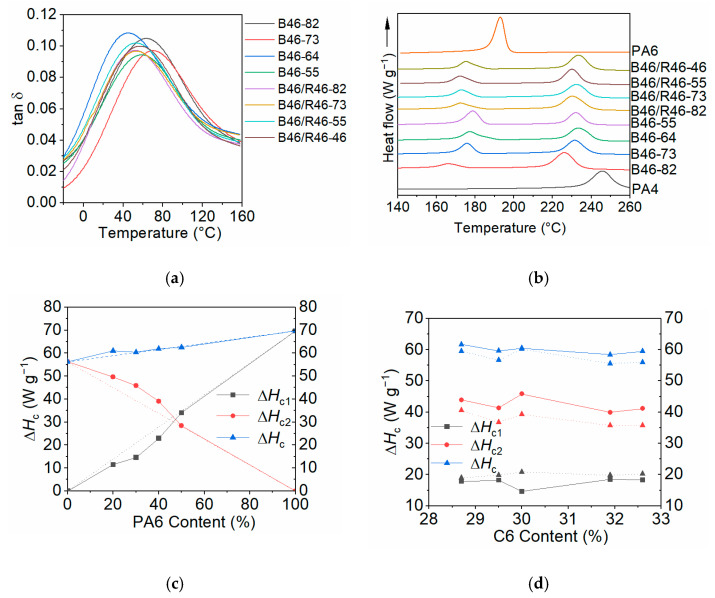
(**a**) Temperature-dependence of the tan δ values of the B46 and B46/R46 blends. (**b**) DSC heat flow curves of the B46 and B46/R46 blends during cooling crystallization. Crystallization enthalpies of the (**c**) B46 and (**d**) B46/R46 blends. Black dotted line: the theoretical crystallization enthalpy (assuming no interactions between the blended components) of PA6; Red dotted line: the theoretical crystallization enthalpy of PA4; Blue dotted line: total theoretical crystallization enthalpy.

The *T*_g_ values of the B46 and B46/R46 blends were determined using DMA, and the loss factor (tan δ, the ratio of the loss modulus to the storage modulus) values of the samples are shown in Figure 4a. The tan δ value indicates the energy absorption capacity of a material. The peak temperature of the tan δ curve corresponds to the *T*_g_ value of the material [39]. The *T*_g_ values are summarized in Table S3. Typically, a single *T*_g_ value indicates that the blend components are miscible at the molecular level [40]. The presence of single tan δ peaks for the B46 and B46/R46 blends in this study confirms the miscibility of PA4, PA6, and R46 in the amorphous state. The SEM images in Figure S3 confirm that all the blends had a homogeneous structure, with no apparent phase separation between the components.

B46-73 exhibited the highest *T*_g_ value among all the B46 blends, which is consistent with it having the highest crystallinity, as listed in Table 2. The data suggest that the higher the crystallinity, the greater the presence of the physical crosslinking points that restrict the segment migration, thus resulting in a higher *T*_g_. However, although B46-64 had the lowest *T*_g_ value, its crystallinity was not the lowest (Table 2). To explain this finding, the MD simulation results shown in Figure 3 were considered. Although the hydrogen bond densities of B46-64 and B46-55 were similar, the degree of orientation of the local molecular chains in B46-64 was lower than that in B46-55, and thus the mobility of the molecular chains in B46-64 was higher. Consequently, the *T*_g_ value of B46-64 was lower than that of B46-55. Further explanation of the *T*_g_ of B46-82 is given in Section 3.2.2. For the B46/R46 blends, B46/R46-46 was found to have the highest *T*_g_ value owing to its high copolymerization structure sequence (S_46_) content, which obstructs chain movement.

#### 3.2.2. Crystallization Behavior

The thermal behavior of the B46 and B46/R46 blends during cooling crystallization was analyzed using DSC. Owing to the thermal decomposition that occurred for the blend with 20% PA6 and considerable crystallization-induced phase separation observed for the blends with 40–50% PA6, the B46-73 blend (with a PA4/PA6 ratio of 70/30 *w*/*w*) was selected to prepare B46/R46 blends with different R46 copolymers. Figure 4b shows the DSC heat flow curves of the B46 and B46/R46 blends during cooling crystallization. The arrow indicates the direction of heat release. The curves contain two exothermic peaks. The low- and high-temperature exothermic peaks, which are downshifted from the exothermic peaks of pure PA6 and PA4, correspond to the crystallization temperatures of the PA6- (*T*_c1_) and PA4-rich (*T*_c2_) phases, respectively.

For the B46 blends, the *T*_c1_ values upshifted gradually with increasing PA6 content. This was attributed to the PA4-rich phase crystallizing first, and the resulting PA4-rich crystals nucleating the PA6-rich crystals. The *T*_c2_ values of the B46 blends downshifted with increasing PA6 content (except for B46-82). PA6 was in the molten state at the crystallization temperature of the PA4-rich phase and served as a diluent. This hindered the regular arrangement of the molecular chains for the PA4-rich phase, which resulted in a decrease in *T*_c2_. The *T*_c2_ value of the B46 blend with 20% PA6 (B46-82) was the lowest. This indicates that blends with high PA4 contents undergo thermal decomposition during processing, thereby decreasing the molecular weight of the blend. The plasticizing role of small-molecule components generated by thermal decomposition resulted in a decrease in the *T*_c2_ and *T*_g_ values of B46-82.

The *T*_c1_ and *T*_c2_ values of the B46/R46 blends were lower than those of B46-73. The melting point of the S_46_-rich phase of R46 was approximately 140–150 °C [29]. Therefore, R46 was still molten at the crystallization temperature of the PA6-rich phase. Consequently, the S_46_-rich phase of R46 served as a diluent, decreasing the concentration of PA6 and thereby preventing the crystallization of the PA6-rich phase. The *T*_c1_ values of all the B46/R46 blends, except B46/R46-46, were comparable and considerably lower than that of B46-73. The *T*_c1_ value of the B46/R46-46 blend was higher than those of the other three B46/R46 blends, indicating that the amount of S_46_ entering the PA6-rich phase was lower than that in the other three B46/R46 blends.

Figure 4c illustrates the changes in the crystallization enthalpies (Δ*H*_c_) of the PA6- and PA4-rich phases of the B46 blends with increasing PA6 content. The dotted lines represent the theoretical crystallization enthalpy (Δ*H*_cT_), assuming no interactions between the blended components (“unmixed blends”). The Δ*H*_cT_ values were calculated using the mass fractions of the blend components and the simple rule of mixtures. For the B46 blends with 20–40% PA6, the Δ*H*_c_ values of the PA4- and PA6-rich phases positively and negatively deviated, respectively, from the Δ*H*_cT_ values. The extent of the deviations decreased with increasing PA6 content. This indicates that, although phase separation occurred during crystallization (as indicated by the presence of two crystallization peaks), a fraction of the PA6 chains participated in the crystallization of the PA4 phase. Accordingly, the Δ*H*_c_ value of the PA4-rich phase was higher than the Δ*H*_cT_ value. At higher PA6 contents, phase separation was favored, which prevented the incorporation of PA6 chains into the crystalline PA4 phase. Crystallization-induced phase separation was particularly pronounced for B46-55 (with 50% PA6); therefore, the Δ*H*_c_ and Δ*H*_cT_ values of B46-55 were similar.

Figure 4d illustrates the changes in the Δ*H*_c_ values of the PA6- and PA4-rich phases of the B46/R46 blends (Δ*H*_c1E_ and Δ*H*_c2E_, respectively) with increasing C6 value of the blends. The total Δ*H*_c_ values of the B46/R46 blends were lower than that of B46-73. When combined with the results shown in Table 2, it is evident that the B46/R46 blends displayed lower crystallinities than B46-73. The black dotted line in Figure 4d represents the theoretical Δ*H*_c_ values of PA6 (Δ*H*_c1T_) assuming an “unmixed” state, as calculated using the blending ratio, and the red dotted line represents the theoretical Δ*H*_c_ values of PA4 (Δ*H*_c2T_), as calculated from the sum of the Δ*H*_c_ values of the “unmixed” state of the PA4 and PA4-rich phases of R46. The Δ*H*_c1E_ value was lower than the Δ*H*_c1T_ value, indicating that homopolymer PA6 was incorporated into the PA4-rich phase, albeit to a lower degree than that in B46-73. This suggests that R46 was more readily incorporated into the PA4-rich phase than homopolymer PA6. The difference between the Δ*H*_c2T_ and Δ*H*_c2E_ values (Δ*H*_c2T_ – Δ*H*_c2E_) for the B46/R46 blends increased with increasing C6 content. B46/R46-82 presented the smallest Δ*H*_c2T_ – Δ*H*_c2E_ value among the B46/R46 blends. The high amount of PA4-rich phase in R46-82 increased the content of PA4 structural units (*C*_4_) in the blend. This can induce thermal decomposition during heating, thereby decreasing the experimental Δ*H*_c_ value and changing the amount of R46 incorporated.

#### 3.2.3. Thermal Stability

**Figure 5:**
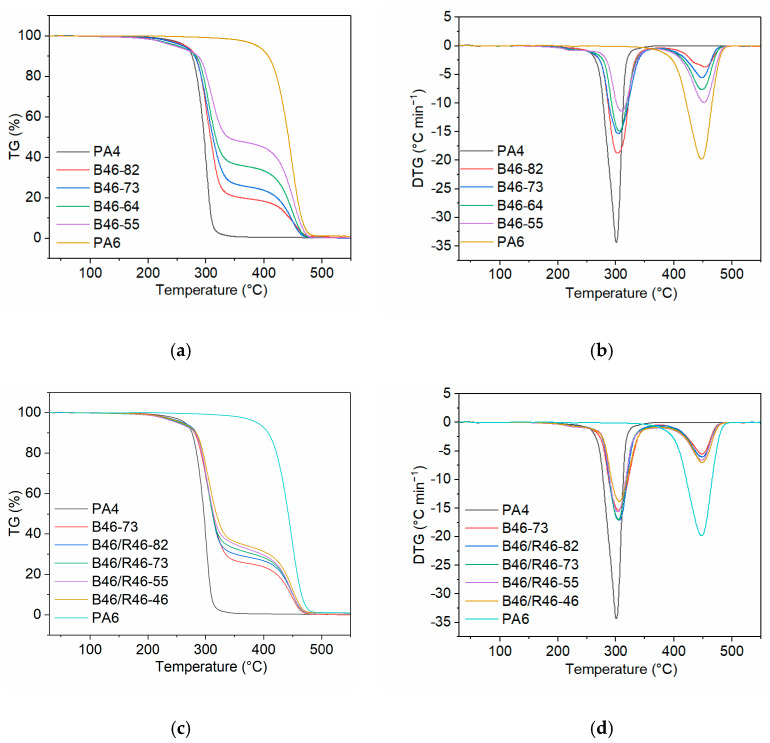
(**a**) TGA and (**b**) DTG curves of the B46 blends. (**c**) TGA and (**d**) DTG curves of the B46/R46 blends.

The thermal stability of the B46 and B46/R46 blends was analyzed using TGA. Figure 5a,b show the TGA and differential thermogravimetry (DTG) curves, respectively, of the B46 blends. The maximum degradation temperatures (*T*_deg_) and the temperatures at which 5%, 10%, and 50% thermal decomposition occurred (*T*_5_, *T*_10_, and *T*_50_, respectively) are listed in Table 3. Considering the *T*_deg_ values of PA4 and PA6 (301.2 and 448.0 °C, respectively), the DTG peaks at 302.5–310.7 °C (*T*_deg1_) and 454.0–451.9 °C (*T*_deg2_) were attributed to the thermal decomposition of the PA4- and PA6-rich phases, respectively.

The slope of the TGA curve for the thermal decomposition of the PA4-rich phase decreased with increasing PA6 content, indicating that the decomposition rate of the PA4-rich phase decreased, and the *T*_10_ and *T*_50_ values increased gradually. These results suggest that PA6 can effectively improve the thermal stability of the blended materials. Figure 5b shows that the *T*_deg1_ value of the B46 blends gradually shifted to a higher temperature with increasing PA6 content. This indicates that the low amide bond density of the PA4-rich phase improved the thermal stability of the blend. Furthermore, the *T*_deg2_ values of the B46 blends were higher than the *T*_deg_ value of pure PA6, indicating that blending improved the thermal stability of the PA6-rich phase. The *T*_5_ value of the B46 blends decreased gradually with increasing PA6 content. This indicates that higher PA6 contents prevented the crystallization of the PA4-rich phase, thereby expanding the amorphous region and causing a decrease in the *T*_5_ value.

Figure 5c,d show the TGA and DTG curves of the B46/R46 blends. With the increase in the C6 content of R46, the decomposition rate of the PA4-rich phase decreased slightly and the *T*_50_ value increased gradually. This indicates that the higher the C6 content, the higher the thermal stability of the blend. The B46/R46 blends all had similar *T*_deg1_ and *T*_deg2_ values (no significant difference) to B46-73. The *T*_5_ value of B46/R46-82 was not significantly different from that of B46-73, and the other B46/R46 blends all had lower *T*_5_ values than B46-73. The WAXD data (Table 2) showed that the crystallinity of B46/R46-82 was higher than those of the other tested B46/R46 blends. This suggests that the amorphous regions of the other B46/R46 blends were larger than those in B46/R46-82, and, accordingly, the *T*_5_ value of B46/R46-82 was higher than those of the other B46/R46 blends.

### 3.3. Mechanical Properties

The tensile strengths, fracture strains, and Young’s modulus of the B46 and B46/R46 blends are shown in Table 4. The typical stress–strain curves of each blend are shown in Figure S4. All blends exhibit the common mechanical behaviors of polyamides. The tensile strengths and fracture strains of the blends were higher than those of the PA4 homopolymer. The tensile strength, fracture strain, and Young’s modulus of B46-82 were lower than those of the other B46 blends, which is likely due to the thermal decomposition that occurred during the melt processing of the B46-82 blend. Such thermal decomposition would decrease the molecular weight of PA4 and the mechanical properties of the B46-82 blend. The tensile strength, fracture strain, and Young’s modulus of B46-55 were lower than those of B46-73 and B46-64. When combined with the data in Figure 4c, we can conclude that crystallization-induced phase separation can also deteriorate the mechanical properties.

The mechanical properties of the B46/R46 blends were inferior to those of all the B46 blends, except for those of B46-82. The grain size and crystallinity of the B46/R46 blends were smaller and lower, respectively, than those of the B46 blends. Studies have shown that Young’s modulus is related to the degree of crystallinity, with a high crystalline fraction corresponding to a high Young’s modulus [41]. Therefore, the changes in the Young’s modulus of the B46 and B46/R46 blends with composition (Table 4) are consistent with the changes in crystallinity (Table 2).

## 4. Conclusions

The effects of composition on the properties and crystallization behavior of B46 and B46/R46 blends were studied. For the B46 blends, owing to crystallization induction, two crystalline phases formed (i.e., the PA4- and PA6-rich phases). As the PA6 content of the B46 blends increased from 20% to 50%, the decomposition rate of the blend decreased, and the *T*_deg1_ value of the PA4-rich phase increased by 10 °C; hydrogen bonding weakened, causing a decrease in crystallinity from 55.1% to 45.1%; the *T*_c_ and *T*_m_ values of the PA4-rich phase increased by 5.9 and 5.2 °C, respectively; and the *T*_c_ and *T*_m_ values of the PA6-rich phase increased by 12.7 and 13.2 °C, respectively. The participation of the PA6 chain segments in the crystallization of the PA4-rich phase diminished gradually. Upon increasing the PA6 content to 50%, crystallization-induced phase separation became evident. PA6-caused dilution suppressed the crystallization of the PA4-rich phase, whereas the pre-crystallization of the PA4-rich phase promoted the nucleation and crystallization of the PA6-rich phase. MD simulations showed that as the PA6 content increased from 20% to 40%, the crystallinity of the blend decreased by 6.8%. The crystallinity of B46-55 was higher than those of B46-82, B46-73, and B46-64. This may be because B46-82, B46-73, and B46-64 have a higher content of PA4 molecular chains than B46-55 and a thus higher hydrogen bond density, which restricts the free movement of the molecular chains. The tensile strengths and fracture strains of the B46 blends were 35–67 MPa and 225–369%, respectively. Therefore, the mechanical properties of the B46 blends were good.

For the B46/R46 blends, the decomposition rate decreased and the thermal stability improved as the *C*6 content of the R46 copolymer increased. Moreover, hydrogen bonding was weakened and crystallinity decreased from 47.9% for B46/R46-82 to 43.7% for B46/R46-46 (7.7–11.9% decrease relative to B46-73). The addition of R46 to B46 decreased the *T*_c_ and *T*_m_ values and the crystallinity of the samples. Moreover, the grain size decreased from 67 Å for B46-73 to 44–48 Å for the B46/R46 blends. R46 was more likely to incorporate within the PA4-rich phase than the PA6 homopolymer was, thereby hindering the two-phase crystallization of the blends. The tensile strengths and fracture strains of the B46/R46 blends were 41–55 MPa and 224–309%, respectively. Therefore, the B46/R46 blends exhibited good mechanical properties.

## Figures and Tables

**Table 1:** Compositions of the B46 and B46/R46 blends.

Sample	PA4/PA6 (*w*/*w*)	R46-82 (wt%) ^1^	R46-73 (wt%)	R46-55 (wt%)	R46-46 (wt%)
B46-82	80/20	-	-	-	-
B46-73	70/30	-	-	-	-
B46-64	60/40	-	-	-	-
B46-55	50/50	-	-	-	-
B46/R46-82	70/30	10	-	-	-
B46/R46-73	70/30	-	10	-	-
B46/R46-55	70/30	-	-	10	-
B46/R46-46	70/30	-	-	-	10

^1^ The R46 content is a percentage of the total mass of the blend.

**Table 2:** Crystallization parameters of the B46 and B46/R46 blends.

Sample	2*θ* (°)	*A*_1_/*A*_2_	Grain Size	Interplanar Spacing (Å)	Crystallinity (%)
B46-82	20.9/23.9	0.06	78	4.24/3.73	55.1
B46-73	20.7/23.7	0.05	67	4.29/3.75	55.6
B46-64	20.6/23.8	0.04	53	4.31/3.73	47.8
B46-55	20.6/23.9	0.04	52	4.31/3.73	45.1
B46/R46-82	20.7/23.8	0.12	48	4.29/3.73	47.9
B46/R46-73	20.7/23.8	0.11	46	4.29/3.73	43.8
B46/R46-55	20.7/23.8	0.10	44	4.30/3.74	44.9
B46/R46-46	20.7/23.8	0.15	48	4.30/3.74	43.7

**Table 3:** Thermal decomposition temperatures of the B46 and B46/R46 blends with various compositions.

Sample	*T*_5_ (°C)	*T*_10_ (°C)	*T*_50_ (°C)	DTG Peak 1		DTG Peak 2	
				*T*_deg1_ (°C)	TG (%)	*T*_deg2_ (°C)	TG (%)
PA4	264.2	276.2	296.5	301.2	35.3	-	-
B46-82	265.0	281.8	307.6	302.5	60.7	454.0	6.5
B46-73	261.2	281.0	312.4	304.6	61.5	448.8	8.9
B46-64	250.8	282.2	317.1	305.9	65.7	448.0	14.0
B46-55	246.5	286.1	338.2	310.7	69.9	451.9	18.3
B46/R46-82	261.6	283.1	311.5	305.9	60.3	449.3	10.4
B46/R46-73	255.1	282.3	311.9	304.6	62.1	448.0	11.4
B46/R46-55	251.3	281.0	313.7	303.8	64.5	448.4	11.5
B46/R46-46	259.4	283.1	318.8	306.4	65.4	448.8	13.3
PA6	389.0	407.9	442.4	-	-	448.0	38.6

**Table 4:** Mechanical properties of the B46 and B46/R46 blends.

Sample	Tensile Strength (MPa)	Fracture Strain (%)	Young’s Moduli (MPa)
PA4	31.99 ± 2.36	112.88 ± 12.35	870.35 ± 13.44
B46-82	35.17 ± 4.97	225.88 ± 51.39	850.16 ± 21.57
B46-73	63.11 ± 15.39	348.72 ± 87.17	921.95 ± 40.35
B46-64	67.40 ± 11.61	369.88 ± 27.37	913.87 ± 20.85
B46-55	56.49 ± 1.70	298.23 ± 40.11	866.49 ± 31.21
B46/R46-82	48.17 ± 2.02	224.09 ± 4.26	825.71 ± 4.56
B46/R46-73	41.30 ± 3.23	262.00 ± 26.04	791.91 ± 19.82
B46/R46-55	51.21 ± 0.08	309.24 ± 26.25	727.95 ± 22.36
B46/R46-46	55.57 ± 4.54	299.31 ± 28.70	797.43 ± 19.64
PA6	71.38 ± 4.42	338.06 ± 20.44	669.38 ± 21.68

## Data Availability

All data generated or analyzed during this study are included in this submitted article.

## Supplementary Materials

The following supporting information can be downloaded at: https://www.mdpi.com/article/10.3390/polym15163399/s1, Computational methods; Figure S1. Schematic of the hydrogen bond in the system; Figure S2. The peak-differentiating and fitting results of B46 and B46/R46 blends; Figure S3. Cross-sectional SEM images of B46 and B46/R46 blends; Figure S4. Stress–strain curves of B46 and B46/R46 blends; Table S1. Molecular chain in the system; Table S2. Molecular chain information of the B46 blends; Table S3. The *T*_g_ values of B46 and B46/R46 blends. References [33,34,35,36,37,38] are cited in Supplementary Materials File.
