# Supplementary material for: Crystallization and Performance of Polyamide Blends Comprising Polyamide 4, Polyamide 6, and Their Copolymers
Source: Polymers (Basel). 2023 Aug 14;15(16):3399. doi: 10.3390/polym15163399 (PMC10459628; doi:10.3390/polym15163399)
Supplement: Supplementary file 1 [file polymers-15-03399-s001.zip › polymers-2554513-supplementary.pdf]

polymers

MDPI

Supplementary Material
Crystallization and Performance of Polyamide Blends Comprising Polyamide 4, Polyamide 6, and Their Copolymers

Yajing Zhang [1,2], Mingda Wang [1,2], Di Zhang [1,2], Yibing Wang [1,2], Li Wang [1,2], Yongjun Qiu [1,2,3], Liquan Wang [4], Tao Chen [2,4,*] and Liming Zhao [1,2,3,*]

[1] State Key Laboratory of Bioreactor Engineering, East China University of Science and Technology, Shanghai 200237, China
[2] Key Laboratory of Biobased Material Engineering, China National Light Industry, East China University of Science and Technology, Shanghai 200237, China
[3] Shanghai Collaborative Innovation Center for Biomanufacturing Technology (SCICBT), Shanghai 200237, China
[4] Shanghai Key Laboratory of Advanced Polymeric Materials, School of Materials Science and Engineering, East China University of Science and Technology, Shanghai 200237, China
* Correspondence: tchen@ecust.edu.cn (T.C.); zhaoliming@ecust.edu.cn (L.Z.)

1. Computational methods

The schematic of PA4 and PA6 molecular chains is shown in Table S1, which used the coarse-grained (CG) Gaussian chain model ("bead-spring chain" model) [1]. Bead A (pink) represents the N atom in the amide group, bead H (grey) represents the H atoms attached to the N atoms, bead B (purple) represents the carbonyl group in the amide group, bead C (green) represents the methylene group in the molecular chain of PA4, and bead D (blue) represents the methylene group in the PA6 molecular chain.

The interactions between atoms in MD are represented by a potential function (force field). The system potential energy (U) in this study is the sum of the nonbonding potential energy (U_{nb}), bond potential energy (U_b), bond-angle-bending potential energy (U_A), and hydrogen-bond interaction potential energy (U_{hb}).

The nonbonding potential energy U_{nb} in the system conforms to the modified Leonard–Jones (LJ) interaction [2]. The bond potential energy U_b is represented by a modified finitely extensible nonlinear elastic potential (FENE) for the FENE potential can only stretch to a limited length, which deviates less from the actual value [3]. The bond angle potential energy U_A is calculated using the harmonic form [4]. The H atom connected to bead A can form a hydrogen bond with bead B (as shown in Figure S1). The hydrogen bond interaction between AHB and the DREIDING force field assumes the same functional form [5].

The Verlet velocity algorithm was used to calculate the equation of motion with a time step of 0.005. The simulation was run under the NPT ensemble while maintaining p^*=1, and T^* was reduced from 2.0 to 0.1 by a drop of 0.1 every 300,000 steps.

To calculate the local orientation degrees of B46, the direction of each bond on the main chain is calculated firstly [4]. The bond orientations were characterized by second-order Legendre polynomials $\langle P_2 \rangle$ [6]. Then the box was divided into 1000 small boxes after removing the H atoms according to the position of each bead in the box. The percentages of the number of small boxes whose $\langle P_2 \rangle$ was higher than 0.7, 0.8, and 0.9 were determined. The crystallinity of the B46 blend system was defined as the percentage of $\langle P_2 \rangle > 0.8$.

2. Figures

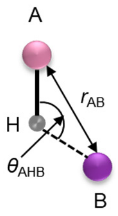

Figure S1. Schematic of the hydrogen bond in the system.

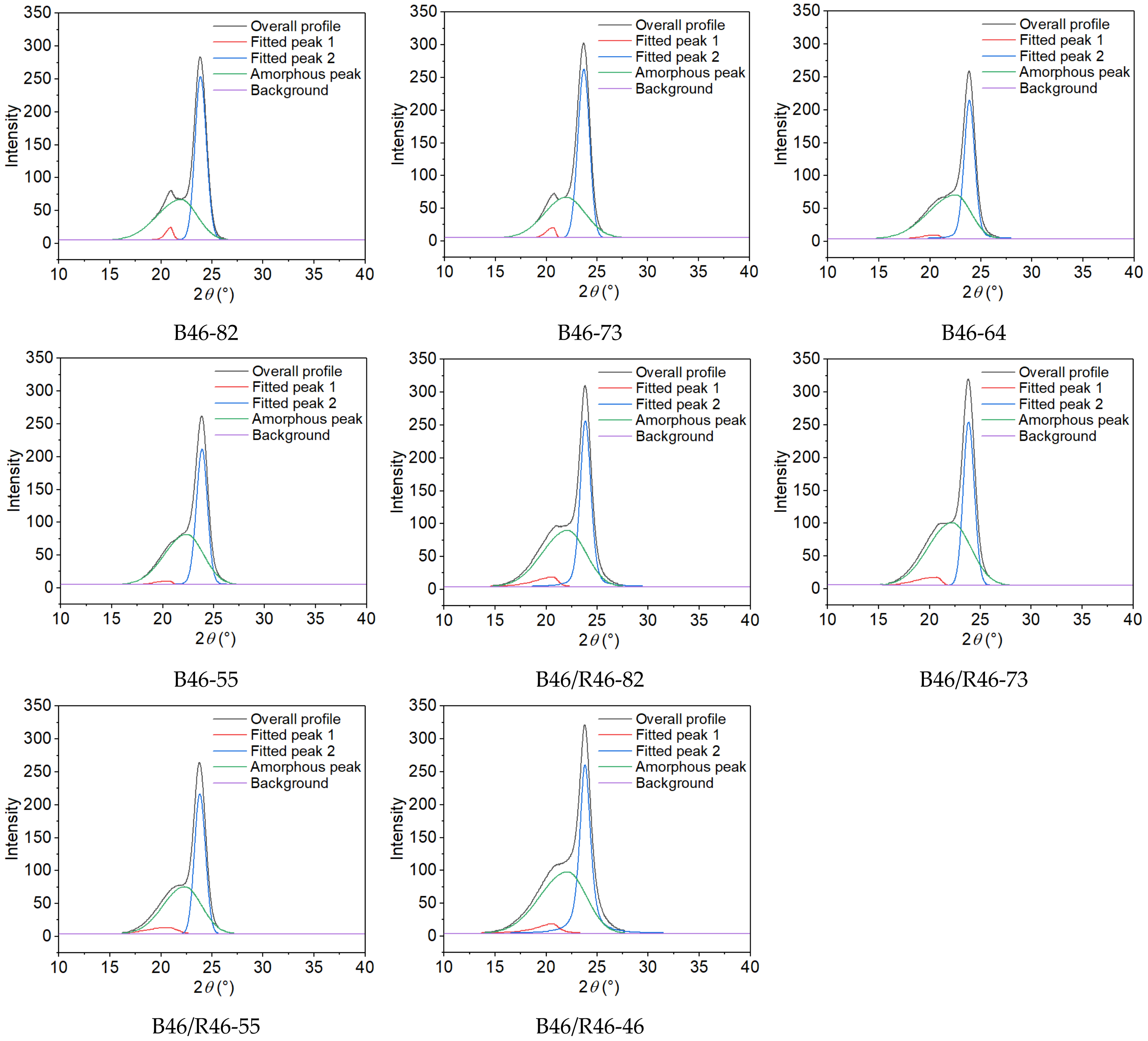

Figure S2. The peak-differentiating and fitting results of B46 and B46/R46 blends.

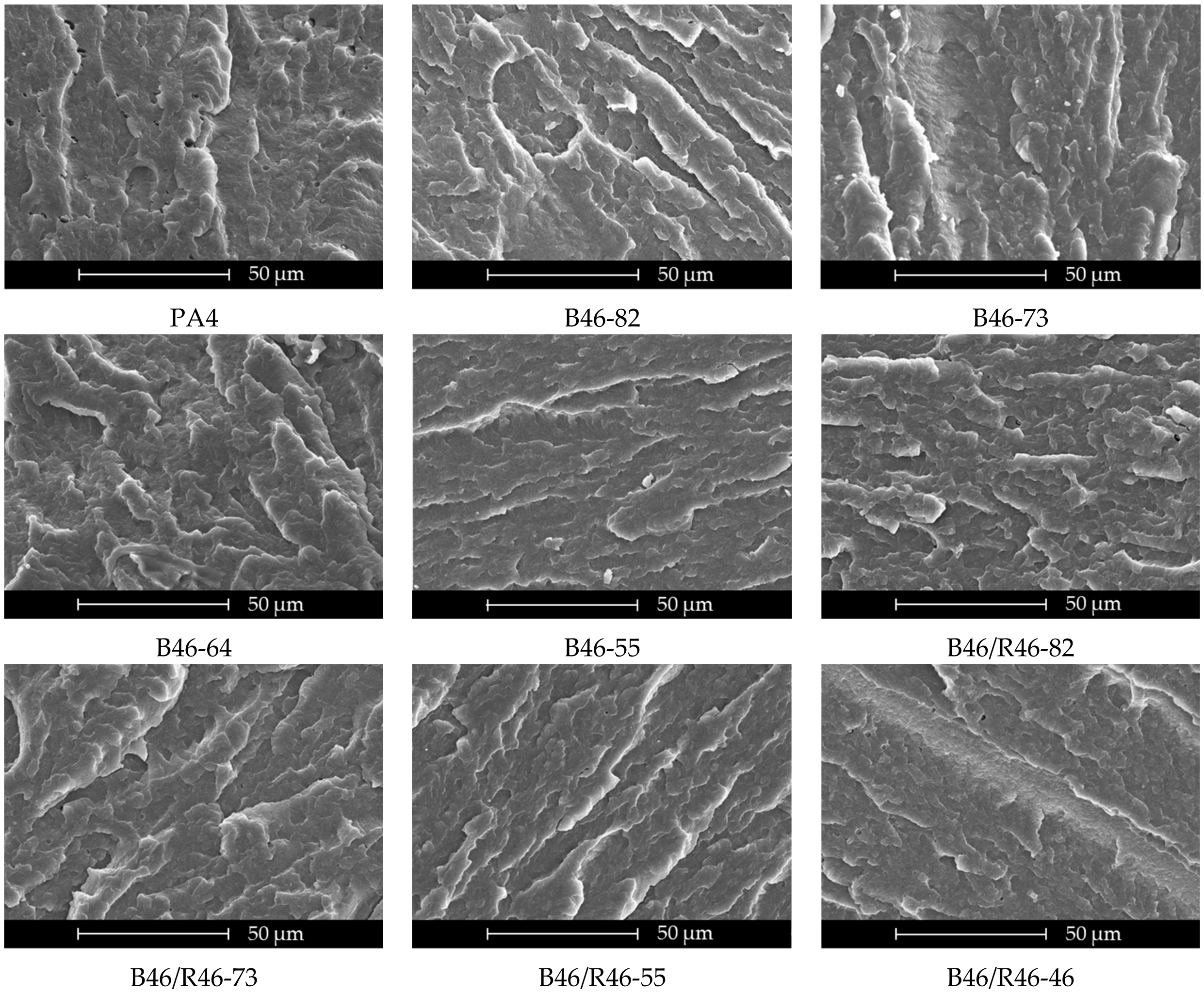

Figure S3. Cross-sectional SEM images of B46 and B46/R46 blends.

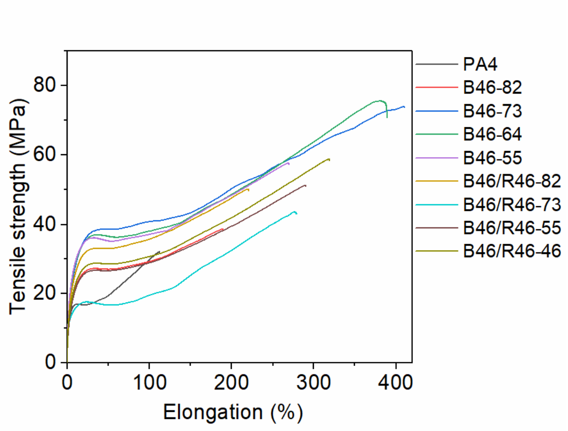

Figure S4. Stress-strain curves of B46 and B46/R46 blends.

3. Tables

Table S1. Molecular chain in the system.

PA	molecular formula	model
PA4	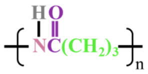	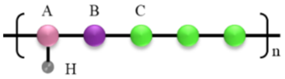
PA6	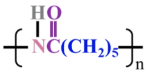	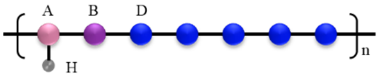

Table S2. Molecular chain information of the B46 blends.

Sample	Segment number (*n*)		Molecular number	
	PA4	PA6	PA4	PA6
B46-82	16	13	160	40
B46-73	16	13	140	60
B46-64	16	13	120	80
B46-55	16	13	100	100

Table S3. The T_g values of B46 and B46/R46 blends.

Sample	T_g (°C)
B46-82	63.3
B46-73	69.3
B46-64	44.6
B46-55	58.1
B46/R46-82	51.6
B46/R46-73	52.9
B46/R46-55	52.7
B46/R46-46	56.7

References

1. Kremer, K.; Grest, G.S. Dynamics of entangled linear polymer melts: A molecular-dynamics simulation. *J. Chem. Phys.* **1990**, *92*, 5057-5086.
2. Yeung, C.; Herrmann, K.A. Molecular dynamics simulation of reactive compatibilization of polymer blends. *Macromolecules* **2003**, *36*, 229-237.
3. Hsieh, C.C.; Jain, S.; Larson, R.G. Brownian dynamics simulations with stiff finitely extensible nonlinear elastic-Fraenkel springs as approximations to rods in bead-rod models. *J. Chem. Phys.* **2006**, *124*, 044911.
4. Hossain, D.; Tschopp, M.A.; Ward, D.K.; Bouvard, J.L.; Wang, P.; Horstemeyer, M.F. Molecular dynamics simulations of deformation mechanisms of amorphous polyethylene. *Polymer* **2010**, *51*, 6071-6083.
5. Hong, W.; Lin, J.P.; Tian, X.H.; Wang, L.Q. Linear and nonlinear viscoelasticity of self-associative hydrogen-bonded polymers. *Polymer* **2021**, *235*, 124301.
6. Liu, J.; Wu, S.Z.; Zhang, L.Q.; Wang, W.C.; Cao, D.P. Molecular dynamics simulation for insight into microscopic mechanism of polymer reinforcement. *Phys. Chem. Chem. Phys.* **2011**, *13*, 518-529.
